# Development and Preliminary Application of a Triplex Real-Time Quantitative PCR Assay for the Simultaneous Detection of *Entamoeba histolytica*, *Giardia lamblia*, and *Cryptosporidium parvum*

**DOI:** 10.3389/fmicb.2022.888529

**Published:** 2022-04-28

**Authors:** Yaoguang Zhang, Jian Chen, Hao Pan, Xiaojiang Ma, Li Jiang, Qian Zhu, Huanyu Wu, Zhenyu Wang

**Affiliations:** ^1^Shanghai Municipal Center for Disease Control and Prevention, Shanghai, China; ^2^Shanghai Institutes of Preventive Medicine, Shanghai, China

**Keywords:** triplex real-time quantitative PCR assay, *Entamoeba histolytica*, *Giardia lamblia*, *Cryptosporidium parvum*, application

## Abstract

**Background:**

The protozoan parasites including *Entamoeba histolytica*, *Giardia lamblia*, and *Cryptosporidium parvum* can infect the human intestinal tract and cause serious diseases. In this study, we aimed to develop a triplex real-time quantitative PCR (qPCR) for the simultaneous differential detection of these three intestinal protozoa.

**Methods:**

Specific primers and TaqMan probes were designed for the 16S-like SSU rRNA sequence of *E. histolytica*, the gdh sequence of *G. lamblia*, and the 18srRNA sequence of *C. parvum*. A triplex qPCR assay was developed based on single-duplicate experiments to evaluate its limit of detection (LOD), specificity, stability, and reproducibility. Additionally, 163 fecal samples from patients with diarrhea who tested positive for copro-antigen were tested to verify the practicality of the assay.

**Results:**

The triplex qPCR assay could specifically detect *E. histolytica*, *G. lamblia*, and *C. parvum* without cross-reactivity amongst the target-specific TaqMan probes of these three intestinal protozoan parasites and did not produce amplification curves for any other non-target species, and had good specificity. Amplification of serial dilutions showed that the triplex qPCR detected as little as 500 copies/μL of standard plasmid DNA. The standard curve displayed good linearity between 5 × 10^2^ and 5 × 10^8^ copies/μL; qPCR assays were performed with an efficiency of more than 95% and *R*^2^ values were greater than 0.99. The triplex qPCR assay had good repeatability with intra- and inter-assay coefficients of variation less than 1.92%. Among the 163 fecal samples, four samples were confirmed to be positive for *C. parvum* using the triplex qPCR assay.

**Conclusion:**

The triplex qPCR established in this study not only provides a rapid, sensitive, specific tool for the simultaneous detection of *E. histolytica*, *G. lamblia*, and *C. parvum*, but also has good practical application value.

## Introduction

The protozoan parasites *Entamoeba histolytica*, *Giardia lamblia*, and *Cryptosporidium* spp. are the causative agents of amebiasis, giardiasis, and cryptosporidiosis, respectively. From 2005 to 2019, a total of 28,229 cases of amoebic dysentery and seven resulting deaths were reported in China (Huang et al., 2020). Annually, 280 million people worldwide are estimated to have clinically diagnosable giardiasis (Feng and Xiao, 2011; Ryan and Cacciò, 2013; Einarsson et al., 2016; Squire and Ryan, 2017). And approximately 28.5 million giardiasis cases are estimated to occur in humans per year in China (Feng and Xiao, 2011; Li et al., 2017). The weighted infection rate of *Entamoeba histolytica* and *Giardia lamblia* was 0.06 and 0.60%, respectively (Zhou, 2018). In China, an average prevalence of 2.97% involving at least 200,054 people from 27 provinces has been revealed in a retrospective epidemiological analysis of human Cryptosporidium infections (1987–2018); the burden of disease caused by *Cryptosporidium* varies between and within areas (Liu et al., 2020). The three intestinal protozoa discussed in this article have very simple biological cycles without intermediate hosts. Infection occurs via the fecal-oral route and these agents can infect the human intestinal tract, causing serious disease (Ghenghesh et al., 2016; Li et al., 2017; Xu et al., 2018).

*E. histolytica* is an internationally recognized pathogenic entamoeba that is invasive in animals and humans and is found in the human colon. It can cause intestinal amoebiasis (e.g., amoebic dysentery) and extraintestinal amoebiasis (e.g., amoebic liver abscess). *E. histolytica* is one of the main pathogens causing diarrhea in patients. Amebiasis is still a major cause of morbidity and mortality in developing countries, and it remains an important public health problem (Smith, 1997; Li et al., 2021).

*G. lamblia* is one of the most common intestinal parasites in the world, affecting approximately 200 million people annually. *Giardia* is transmitted via the fecal to oral route, most often by ingestion of contaminated food or water (Einarsson et al., 2016). Symptoms of *Giardia* infection include foul-smelling diarrhea, abdominal cramping, bloating, gas, and nausea. Immunocompromised individuals and undernourished children from developing countries are more susceptible to serious manifestations of untreated *Giardia* infection (Júlio et al., 2012; Bartelt and Sartor, 2015).

*C. parvum* is a specialized intracellular protozoan parasite that has a monoxenous life cycle (Zhu and Su, 2018). Infection takes place via oral ingestion of oocysts containing invasive sporozoites. These sporozoites enter intestinal epithelial cells and form a parasitophorous vacuole that is located at the apical part of the host cell, just underneath the brush border (Hemphill et al., 2019). This infection may cause diarrhea and abdominal pain in the host; the course of the disease is mostly self-limiting. Patients with immunodeficiency are more prone to developing severe symptoms and are more often complicated by extraintestinal cryptosporidiosis. Cryptosporidiosis is considered one of the original AIDS-defining illnesses and a major risk factor for mortality, especially compared with other AIDS-defining illnesses (Ahmadpour et al., 2020).

Currently, microscopic examination of stool specimens remains the primary method for diagnosing intestinal protozoan parasites, especially in developing countries. Light microscopy is its most commonly used detection method. However, microscopy can lead to incorrect results, with harmless parasites being interpreted as disease-causing, or life-threatening parasites being missed (Halligan et al., 2014). Many protists are only present in small quantities in fecal samples. Additionally, the quality of the microscopic examination is highly dependent on the skills of the laboratory technician (Khare et al., 2014). There are several PCR-based methods that are used in the diagnosis of *E. histolytica*, *G. lamblia*, and *C. parvum* (Friesen et al., 2018; Tsui et al., 2018; Jerez Puebla et al., 2020; Robinson et al., 2020; Shahbazi et al., 2020; Cai et al., 2021; Calle-Pacheco et al., 2022). Among the PCR methods developed for better diagnostics, real-time quantitative PCR (qPCR) methods are considered the leading ones as they are highly sensitive and specific. To the best of the authors’ knowledge, a few studies refer to use of multiplex qPCR commercial kits for these three protozoan parasites (Laude et al., 2016; Argy et al., 2022). However, these kits have national or regional limitations and are not available in some countries, including China. However, some key parameters, such as sequences of the specific primers and probes or reaction conditions, cannot be disclosed due to commercial confidentiality. Thus, the purpose of this study was to establish a TaqMan-based triplex qPCR assay for the simultaneous detection of *E. histolytica*, *G. lamblia*, and *C. parvum*.

## Materials and Methods

### Parasite Samples and Patient Samples

Parasite samples of *E. histolytica*, *G. lamblia*, *C. parvum*, *C. baileyi*, *Taenia saginata*, *T. solium*, *Clonorchis sinensis*, *Paragonimus westermani*, *Ascaris lumbricoides*, *Plasmodium ovale*, *Leishmania infantum*, *Toxoplasma gondii* and *Entamoeba coli* were obtained during routine testing and stored in the Shanghai Municipal Center for Disease Control and Prevention parasite laboratory.

In total, 163 stool specimens of clinical patients with positive results in immunological assays were collected from the assigned intestinal protozoan-monitoring hospital during the period from 2016 to 2020. The presence of antigens of *E. histolytica*, *C. parvum*, and *G. lamblia* in stool samples from these patients was determined using the RIDA ^®^QUICK *Cryptosporidium/Giardia* Combi test (R-Biopharm AG, Germany) and RIDA ^®^QUICK Entamoeba test (R-Biopharm AG).

### Design of Primers and Probes

Three pairs of specific primers and corresponding TaqMan probes were designed targeting the *E. histolytica* 16S-like SSrRNA gene (GenBank Accession number X56991.1), *G. lamblia* gdh gene (GenBank Accession number KM190761.1), and *C. parvum* 18SrRNA gene (GenBank Accession number NC_006987.1). The probes and primers were designed by using Primer Express 3.0.1 software, Applied Biosystems, United States. The specificity of the probes and primers to their target DNA region was confirmed *in silico* by performing BLAST and Primer-BLAST searches. Nucleotide sequences for the primers and probes described in this study are shown in Table 1. The primers and probes were synthesized by Shanghai BioGerm, Shanghai, China.

**TABLE 1:** Primers and TaqMan probes for quantitative PCR (qPCR).

Target species	Gene	Primer/probe	Sequence(5’-3’)	Length/bp
*Entamoeba histolytica*	16S	Emh-F	GGAAGCATTCAGCAATAACA GGTC	149
		Emh-R	TCGGTACACCACTCACTATC CTTA	
		Emh-FamP	TTAGACATCTTGGGCCGCAC GCGC	
*Giardia lamblia*	GDH	Gla-F	GGACAGTACAAGCGCC TGAG	135
		Gla-R	GTCCTTGCACATCTCCT CCAG	
		Gla-vicP	AGTTCACAGGCGTCCTCAC AGGCAAGA	
*Cryptosporidium parvum*	18S	CryP-F	CGGGGAATTAGGGTT CGATTC	102
		CryP-R	CCTCCCTGTATTAGGATT GGGTAA	
		CryP-1cy5P	ACGGCTACCACATCTAAGGA AGGCAGC	

### DNA Extractions

DNA extractions were performed using the QIAamp DNA Mini Kit and QIAamp DNA Fast Stool Mini Kit (Qiagen, Germany), according to the manufacturer’s instructions. The extracted DNA was stored at −20°C until needed.

### Construction of Standard Plasmids and Validation

The target fragments of the *E. histolytica* 16S-like SSrRNA gene, *G. lamblia* gdh gene, and *C. parvum* 18SrRNA gene were cloned into the PUC19 vector and three plasmids were constructed by a commercial company (Shanghai BioGerm, Shanghai, China). After validation with sequencing (Sangon Biotech, Shanghai, China), the plasmids were, respectively, named Emh, Gla, and CryP. The copy number of the recombinant plasmids was calculated according to the following formula: Copy Number (Copies/μl) = Concentration (g/μl)/(660 × DNA length) × NA (NA: Avogadro’s constant). The concentrations of the recombinant plasmids Emh, Gla, and CryP were adjusted to 5 × 10^8^ copies/μL using sterile distilled water. Plasmids were stored at −20°C until being used as the standard plasmids.

### Optimization of Reaction Parameters of Triplex Quantitative PCR

To optimize the reaction parameters of the triplex qPCR, we changed not only the concentrations of the primers and probes but also the annealing temperature. The triplex qPCR reaction mixture with a total volume of 25 μL included Premix Ex Taq (Probe qPCR) 12.5 μL, 2 μL of a mixture of three standard plasmids (with 5 × 10^5^ copies/μL of each plasmid), three mixed pairs of primers, three probes of different volumes, and sterile distilled water to a final volume of 25 μL. The triplex qPCR conditions were as follows: 5 min at 95°C (initial denaturation), 40 cycles at 95°C for 10 s (denaturation), and 55°C for 40 s (annealing and extension). Using a variety of combinations to test the reaction conditions, we obtained the following parameters: the annealing temperature ranged from 55 to 60°C; the volume of primers (with 20 μmol/L of each primer) was, respectively, 0.25, 0.5, 0.75, 1.00, 1.25, and 1.5 μL; the volume of probes (with 10 μmol/L of each probe) was, respectively, 0.25, 0.5, 0.75, 1.00, 1.25, and 1.5 μL. The final concentrations of the primers, probes, and the amplification conditions were optimized to obtain the maximum ΔRn and minimal cycle threshold (Ct) using the standard plasmids at different dilutions as a template. With the three mixed standard plasmids as templates, the final concentrations of primers and probes and amplification conditions were optimized to obtain the maximum ΔRn and minimum Ct value.

### Construction of Standard Curves of Singleplex and Triplex Quantitative PCR

Standard curves were performed using the recombinant plasmids Emh, Gla, and CryP (5 × 10^2^ to 5 × 10^8^ copies/μL), respectively. The amplifications were carried out with optimized reaction conditions. The assays were repeated three times within the study.

### Limit of Detection of Singleplex and Triplex Quantitative PCR Assays

The standard plasmids Emh, Gla, and CryP with a final concentration of 5 × 10^8^ copies/μL for each plasmid were serially diluted (10-fold dilutions) and then used to determine the LOD of singleplex and triplex qPCR assays. Amplifications were performed with the optimized reaction conditions.

### Specificity Testing of Triplex Quantitative PCR

To validate the specificity of the developed triplex qPCR assay, we used DNA of *E. histolytica*, *G. lamblia*, *C. parvum*, *C. baileyi*, *T. saginata*, *T. solium*, *Clonorchis sinensis*, *Paragonimus westermani*, *Ascaris lumbricoides*, *Plasmodium ovale*, *Leishmania infantum*, *Toxoplasma gondii*, and *Entamoeba coli* for amplification as templates. In the specificity test, sterile distilled water was used as the blank control, normal human fecal DNA as the negative control, and a mixture of three standard plasmids (with 5 × 10^6^ copies/μL of each plasmid) as the positive control.

### Repeatability Testing of Triplex Quantitative PCR

We conducted successive 10-fold serial dilutions of the Emh, Gla, and CryP standard plasmids from 10^3^ to 10^5^ copies/μL to calculate the intra- and inter-assay coefficients of variation (CV) in Ct values for the triplex qPCR assay.

### Co-infection Models Detection of Triplex Quantitative PCR

We mixed two or three standard plasmids in different proportions for co-infection models detection.

### Testing Patients Samples Using Triplex Quantitative PCR

Fecal samples of patients with diarrhea were collected from designated hospitals in Shanghai from 2016 to 2020 and tested for copro-antigen using the RIDA ^®^QUICK *Cryptosporidium/Giardia* Combi test and RIDA ^®^QUICK Entamoeba test (R-Biopharm AG). A total of 163 samples with positive copro-antigen test results were initially screened and numbered. Nucleic acid from the fecal samples was then extracted separately as templates and tested using the triple qPCR assay developed in this study to evaluate the practical application value of this method.

### Statistical Analysis

PCR amplification efficiencies (%) were calculated using the following formula: $E=\left[10^{-\frac{1}{slope}}-1\right]\times 100$. The parameters of standard curves (slope, linearity, and efficiency), standard deviation, and coefficient of variation were calculated by Microsoft Excel 2019.

## Results

### Optimal Reaction System and Conditions of Triplex Quantitative PCR

After optimization, the reaction system with a total volume of 25 μL for the triplex qPCR was as follows: Premix Ex Taq (Probe qPCR) 12.5 μL, 2 μL of template, 0.60 μL each of Emh-F/R and Gla-F/R (20 μmol/L) and 0.5 μL each of the corresponding probes (10 μmol/L), 1.25 μL each of CryP-F/R (20 μmol/L) and 1.25 μL of the probe (10 μmol/L), and sterile distilled water 3.35 μL. The triplex qPCR conditions were as follows: 5 min at 95°C (initial denaturation), 40 cycles at 95°C for 10 s (denaturation), and 55°C for 40 s (annealing and extension).

### Standard Curves of Singleplex and Triplex Quantitative PCR Assays

**FIGURE 1:**
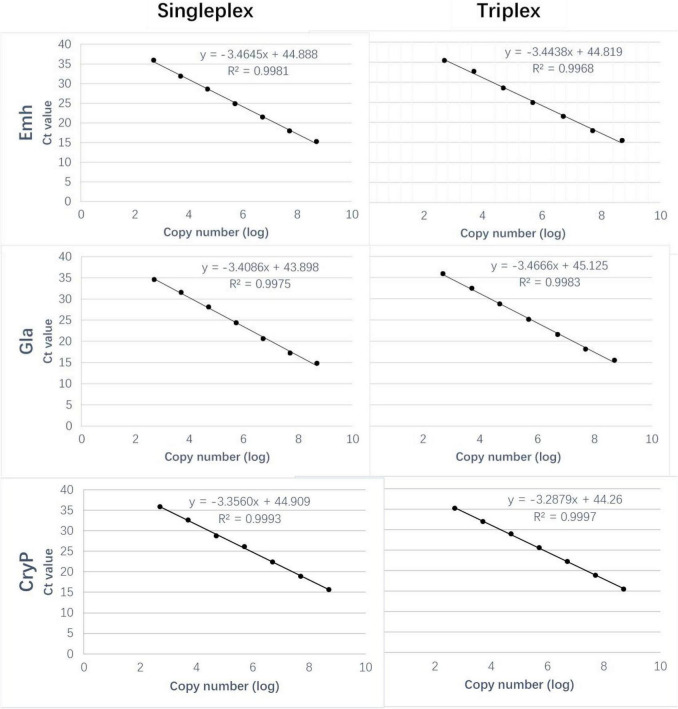
Comparison of the standard curves of the singleplex and triplex Quantitative PCR (qPCR) assays. Ct, cycle threshold.

Standard curves of singleplex and triplex qPCR generated for the three standard plasmids under triplex conditions showed perfect linearity (*R*^2^ > 0.99, Figure 1 and Table 2). Amplification efficiencies for Emh, Gla, and CryP of singleplex qPCR were 94.38, 94.30, and 101.44%, respectively. Amplification efficiencies for Emh, Gla, and CryP of triplex qPCR were 95.94, 96.51, and 98.60%, respectively (Table 2). The results revealed that singleplex and triplex qPCR could efficiently detect the target genes of the three intestinal protozoan parasites. All standard curves had good correlation coefficients and amplification efficiencies, which indicated that the developed qPCR assays were effective.

**TABLE 2:** Parameters of the standard curves of the singleplex and triplex Quantitative PCR (qPCR) assays.

Parameter	Singleplex			Triplex		
	Emh	Gla	CryP	Emh	Gla	CryP
Slope	−3.4645	−3.4086	−3.3560	−3.4438	−3.4666	−3.2879
Linearity (*R*^2^)	0.9981	0.9975	0.9993	0.9968	0.9983	0.9997
Efficiency (%)	94.38	94.30	101.44	95.94	96.51	98.60
Limit of detection (copies/μl)	500	500	500	500	500	500

### Limit of Detection of Singleplex and Triplex Quantitative PCR Assays

The LODs of singleplex and triplex qPCR were all 5 × 10^2^ copies/μL for these three intestinal protozoa (Figure 1 and Table 2). The standard deviation (SD) values were analyzed in triplicate by singleplex and triplex qPCR, which showed that the results of the assays were reliable and accurate (Table 3).

**TABLE 3:** Limit of detection (LOD) and standard curves of singleplex and triplex quantitative PCR (qPCR) assays.

Number of DNA copies (copies/μL)	Singleplex qPCR Ct value (mean ± SD)			Triplex qPCR Ct value (mean ± SD)		
	Emh	Gla	CryP	Emh	Gla	CryP
5 × 10^8^	15.33 ± 0.04	14.81 ± 0.04	15.73 ± 0.81	15.46 ± 0.02	15.51 ± 0.01	15.51 ± 0.20
5 × 10^7^	17.87 ± 0.11	17.23 ± 0.04	18.96 ± 0.19	17.91 ± 0.05	18.15 ± 0.06	18.95 ± 0.13
5 × 10^6^	21.42 ± 0.14	20.65 ± 0.07	22.44 ± 0.18	21.48 ± 0.09	21.59 ± 0.01	22.32 ± 0.17
5 × 10^5^	24.80 ± 0.18	24.39 ± 0.02	26.10 ± 0.25	24.89 ± 0.07	25.16 ± 0.02	25.63 ± 0.17
5 × 10^4^	28.44 ± 0.34	28.10 ± 0.31	28.84 ± 0.13	28.54 ± 0.09	28.77 ± 0.05	28.96 ± 0.21
5 × 10^3^	32.47 ± 0.34	31.56 ± 0.83	32.59 ± 0.18	32.68 ± 0.25	32.49 ± 0.33	32.02 ± 0.24
5 × 10^2^	35.45 ± 0.75	34.58 ± 1.46	35.83 ± 1.16	35.39 ± 0.97	35.91 ± 0.98	35.27 ± 0.40

*SD, standard deviation.*

### Specificity of Triplex Quantitative PCR

**FIGURE 2:**
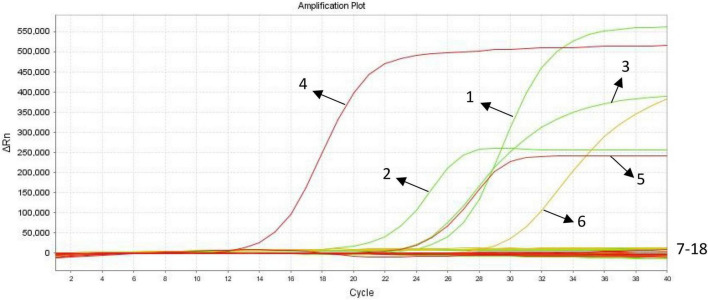
Specificity analysis of triplex quantitative PCR (qPCR). (1) *E. histolytica* positive control(plasmid DNA Emh); (2) *G. lamblia* positive control(plasmid DNA Gla); (3) *C. parvum* positive control(plasmid DNA CryP); (4) *E. histolytica* positive control (genomic DNA); (5) *G. lamblia* positive control(genomic DNA); (6) *C. parvum* positive control(genomic DNA); (7–18) blank control, negative control, *Cryptosporidium baileyi*, *Toxoplasma gondii*, *Clonorchis sinensis*, *Leishmania infantum*, *Ascaris lumbricoides*, *Paragonimus westermani*, *Taenia saginata*, *Taenia solium*, *Plasmodium ovale*, *Entamoeba coli.*

The results were positive for *E. histolytica*, *G. lamblia*, *C. parvum* samples, the standard plasmid, and negative for the blank control, negative control, and other parasite samples (Figure 2) indicating that the developed triplex qPCR assay was sufficiently specific to satisfy the detection requirements.

### Repeatability of Triplex Quantitative PCR

The results of the triplex qPCR intra- and inter-assay showed that the CV of Ct values for each dilution of Emh, Gla, and CryP was less than 2% (Table 4). This indicated that the triplex qPCR had good reproducibility.

**TABLE 4:** Repeatability analysis of triplex quantitative PCR (qPCR).

Species	Copies/μL	Ct values of intra-assay		Ct values of inter-assay	
		x̄ ± s	CV(%)	x̄ ± s	CV(%)
*E. histolytica*	1 × 10^5^	29.65 ± 0.09	0.31	30.20 ± 0.33	1.09
	1 × 10^4^	33.09 ± 0.45	1.36	33.51 ± 0.50	1.48
	1 × 10^3^	36.36 ± 0.62	1.72	36.63 ± 0.43	1.16
*G. lamblia*	1 × 10^5^	25.85 ± 0.08	0.29	26.63 ± 0.27	1.00
	1 × 10^4^	29.19 ± 0.12	0.40	29.76 ± 0.34	1.15
	1 × 10^3^	31.87 ± 0.19	0.60	32.56 ± 0.62	1.92
*C. parvum*	1 × 10^5^	28.59 ± 0.24	0.83	28.62 ± 0.34	1.18
	1 × 10^4^	31.78 ± 0.27	0.86	31.67 ± 0.40	1.25
	1 × 10^3^	34.49 ± 0.17	0.48	34.63 ± 0.59	1.69

*SD, standard deviation; CV, coefficient of variation.*

### Co-infection Models Detection of the Triplex Quantitative PCR

As shown in Table 5, the method could detect two or three protozoan parasites at the combinations of different concentrations. Furthermore, the SD of the co-infection was less than 1%.

**TABLE 5:** The detection of the co-infection models by triplex quantitative PCR (qPCR).

Co-infection proportion*^a^*	Number of DNA copies (copies/μL)			Co-infection real-time PCR Ct Value (mean ± SD)		
	Emh	Gla	CryP	Emh	Gla	CryP
Emh: Gla: CryP = 100:1:1	1 × 10^8^	1 × 10^6^	1 × 10^6^	14.45 ± 0.13	20.51 ± 0.09	20.64 ± 0.93
Emh: Gla: CryP = 10:100:1	1 × 10^7^	1 × 10^8^	1 × 10^6^	17.51 ± 0.57	17.61 ± 0.18	21.09 ± 0.64
Emh: Gla: CryP = 10:1:1	1 × 10^8^	1 × 10^7^	1 × 10^7^	14.04 ± 0.30	21.15 ± 0.50	20.88 ± 0.10
Emh: Gla: CryP = 1:1:10	1 × 10^7^	1 × 10^7^	1 × 10^8^	17.28 ± 0.37	20.57 ± 0.11	16.85 ± 0.48
Emh: Gla: CryP = 1:10:100	1 × 10^6^	1 × 10^7^	1 × 10^8^	20.77 ± 0.37	19.82 ± 0.08	16.73 ± 0.71
Emh: Gla: CryP = 1:1:1	1 × 10^5^	1 × 10^5^	1 × 10^5^	29.65 ± 0.09	25.85 ± 0.08	28.59 ± 0.24
Emh: Gla: CryP = 10:100:1	5 × 10^3^	5 × 10^4^	5 × 10^2^	30.30 ± 0.28	27.33 ± 0.49	36.10 ± 0.28
Emh: Gla = 100:1	1 × 10^8^	1 × 10^6^	–	14.87 ± 0.34	20.02 ± 0.03	–
Gla: CryP = 1:100	–	1 × 10^6^	1 × 10^8^	–	19.89 ± 0.09	17.39 ± 0.27
Emh: CryP = 1:100	1 × 10^6^	–	1 × 10^8^	21.08 ± 0.50	–	17.23 ± 0.63
Emh: CryP = 10:1	1 × 10^7^	–	1 × 10^6^	17.24 ± 0.25	–	20.19 ± 0.28
Emh: Gla = 1:10	1 × 10^6^	1 × 10^7^	–	21.03 ± 0.99	20.70 ± 0.12	–
Gla: CryP = 10:1	–	5 × 10^3^	5 × 10^2^	–	30.54 ± 0.27	36.03 ± 1.00
Emh: CryP = 10:1	5 × 10^3^	–	5 × 10^2^	31.10 ± 0.50	–	35.98 ± 0.74
Emh: Gla = 1:1	5 × 10^2^	5 × 10^2^	–	35.05 ± 0.96	33.25 ± 0.70	–

*^a^There were two kinds of co-infection models included: the triplex co-infection and duplex co-infection.*

*SD, standard deviation.*

*–: represents no design in the corresponding system.*

### Practical Application of Triplex Quantitative PCR

Practical application of the triplex qPCR assay was evaluated in a total of 163 fecal samples. The results showed that four samples were positive for *C. parvum*; the results of the copro-antigen assay for these four samples were also positive for *Cryptosporidium*. The rest of the samples were negative, despite the results of the copro-antigen assay being positive.

## Discussion

Amebiasis, giardiasis, and cryptosporidiosis are difficult to prevent and control because of their zoonotic characteristic and large number of reservoir hosts (Ghenghesh et al., 2016; Li et al., 2017, 2021; Xu et al., 2018). The ability to rapidly detect *E. histolytica*, *G. lamblia*, and *C. parvum* is important in laboratory diagnosis because these pathogens cause serious diseases (Helmy et al., 2014; Hemphill et al., 2019; Li et al., 2020). PCR-based molecular diagnostic techniques have obvious advantages in the accurate identification of intestinal protozoan species and detection of mixed infections of multiple intestinal protozoa. These techniques can compensate for the lack of sensitivity and high skill requirements of microscopic examination (Friesen et al., 2018; Tsui et al., 2018; Jerez Puebla et al., 2020; Robinson et al., 2020; Shahbazi et al., 2020; Cai et al., 2021; Calle-Pacheco et al., 2022). In particular, the qPCR method does not require electrophoresis and the results can be analyzed directly using images and data such as amplification curves and Ct values, making it ideal for use in a variety of medical settings where specialist parasitological microscopy staff are scarce. With the current global COVID-19 pandemic and the need for nucleic acid detection, the coverage of qPCR instruments is increasing at all levels in medical institutions throughout China and worldwide. Therefore, the acceptance of qPCR assays at these institutions is likely to be high.

A number of single or duplex qPCR assays have also been established for *E. histolytica*, *G. lamblia*, or *C. parvum* (Helmy et al., 2014; Tsui et al., 2018; Li et al., 2020; Robinson et al., 2020; Cai et al., 2021; Calle-Pacheco et al., 2022). In this study, a triplex qPCR assay was developed that allows amplification of three target sequences in one reaction using three pairs of primers and probes to simultaneously identify these three target sequences. The sensitivity and specificity of the triplex qPCR assays are determined by the primers and probes. So, it is particularly important to find the optimal primers and probes. We designed the primers and probes in the conservative region of each three intestinal protozoa (Table 1), the specificity of the primers and probes were verified by BLAST and Primer-BLAST. We optimized the annealing temperature, primer and probe concentration to make sure that the developed assay had the greatest amplification efficiency and the lowest Ct value.

In this study, the specificity of each of the Emh, Gla, and CryP triplex qPCRs was evaluated individually against the DNAs from other two protozoa species. In addition, the specificity of each of the three triple qPCRs was evaluated in triplex formats against the DNAs from other intestinal parasites such as *C. baileyi*, *T. saginata*, *T. solium*, *Clonorchis sinensis*, *Paragonimus westermani*, *Ascaris lumbricoides*, and *Entamoeba coli* (*Clonorchis sinensis* and *Paragonimus westermani* are not intestinal parasites, but pathogens can be detected in human fecal samples). The specific primers and probes for these three intestinal protozoa parasites did not exhibit cross-reactivity among them or among any of the seven human parasite species described above. That is to say, the developed assay has good specificity. The standard curves showed good linear correlation between the Ct values and logarithm of concentrations of the three standard plasmids. According to the results, the LOD of the triplex qPCR for the detection of Emh, Gla, and CryP was up to 500 copies/μL.

The results of the repeatability testing of the triplex qPCR revealed that the intra-assay CV and inter-assay CV were both below 2%. Consequently, this may indicate that the triplex assay has a high repeatability (intra-assay) and reproducibility (inter-assay) within the range of detection (Table 4). Noteworthy, in clinical practice, there exists the possibility of mixed infections of different parasites at different concentrations in fecal samples (Hemphill et al., 2019). Thus, the co-infection models detection was used for the determination of the detection efficiency of mixed infection. The result showed that these three intestinal protozoa at different concentrations with different combination could be determined, and the standard deviation of the co-infection detection was less than 1% (Table 5). Therefore, the developed triplex qPCR assay is highly reliable and accurate, and it can also increase throughput while reducing reaction costs by detecting these three intestinal protozoan parasites in just one amplification.

Clinical fecal samples were tested using the triplex qPCR assay developed in this study, and the results were positive for *C. parvum* in four samples and positive for *Cryptosporidium* in the copro-antigen assays. The PCR results of the remaining 159 fecal samples were negative. According to some scholars (Weitzel et al., 2006; Helmy et al., 2014; Uppal et al., 2014; Jerez Puebla et al., 2020), the results of immunological methods are not always accurate. It has also been pointed out that the reliability and reproducibility of results from only immunological assays are controversial (Danišová et al., 2018). Few commercial kits for the diagnosis of intestinal protozoan copro-antigens have been reported in China (Chen et al., 2012; Wang et al., 2021). Furthermore, the results of copro-antigen testing are susceptible to many factors, such as the length of time the samples are left after collection, storage, and transportation conditions. Thus, the role of the copro-antigen assays in this study was to initially screen fecal samples from individuals with suspected intestinal protozoan infections, to appropriately narrow the scope of nucleic acid detection, and to reasonably lessen the workload of laboratory staff. However, the triplex qPCR assay simultaneously amplified well one sample of *E. histolytica*, *G. lamblia*, and *C. parvum* (Figure 2), and the amplification curves of these samples showed significant exponential growth, indicating that the method can be initially applied for the laboratory detection of intestinal protozoa.

In summary, the triplex qPCR assay established in this study is highly sensitive, specific, stable, and repeatable, and it can be used for rapid, specific, and accurate quantitative detection of three important human intestinal protozoa, providing technical support for disease control and medical institutions. In future research, we will strengthen the combined application of molecular biologic, immunologic, and pathogenetic diagnostic methods for these three protozoa, which will help to improve the detection rate and epidemiological surveillance of intestinal protozoa to promote the prevention and control of intestinal protozoiasis.

## Data Availability Statement

The authors acknowledge that the data presented in this study must be deposited and made publicly available in an acceptable repository, prior to publication. Frontiers cannot accept a manuscript that does not adhere to our open data policies.

## Ethics Statement

The studies involving human participants were reviewed and approved by the Health Ethics Committee of Shanghai Municipal Center for Disease Control and Prevention (ref 2019-23). Written informed consent for participation was not required for this study in accordance with the national legislation and the institutional requirements.

## Author Contributions

ZW and HW conceived the research project. YZ, ZW, and QZ performed the experiments. YZ and ZW performed the data analysis. JC, HP, XM, LJ, YZ, and ZW interpreted the data and discussed the results. YZ, ZW, and JC wrote the manuscript. All authors contributed to the article and approved the submitted version.

## Conflict of Interest

The authors declare that the research was conducted in the absence of any commercial or financial relationships that could be construed as a potential conflict of interest.

## Publisher’s Note

All claims expressed in this article are solely those of the authors and do not necessarily represent those of their affiliated organizations, or those of the publisher, the editors and the reviewers. Any product that may be evaluated in this article, or claim that may be made by its manufacturer, is not guaranteed or endorsed by the publisher.
